# Predictors of distant metastatic recurrence in intermediate‐risk papillary thyroid carcinoma

**DOI:** 10.1002/wjs.12289

**Published:** 2024-08-26

**Authors:** Naoyoshi Onoda, Yasuhiro Ito, Akihiro Miya, Minoru Kihara, Akira Miyauchi

**Affiliations:** ^1^ Department of Surgery Kuma Hospital Kobe Japan

**Keywords:** distant metastasis, intermediate‐risk papillary thyroid carcinoma, predictors

## Abstract

**Background:**

Patients with intermediate‐risk papillary thyroid carcinoma (PTC) have a favorable prognosis with standard treatment of total thyroidectomy (TT) and adjuvant radioactive iodine therapy (RAIT). However, the benefits of TT or adjuvant RAIT remain undetermined, and they are often omitted in Japan. We investigated risk factors for life‐threatening distant recurrence in patients with intermediate‐risk PTC who are optimal candidates for adjuvant RAIT.

**Patients and Methods:**

Outcomes without RAIT were retrospectively examined in 4030 intermediate‐risk conventional PTC cases underwent initial surgery from 2005 to 22 (IRB approval 20200709‐1).

**Results:**

Lobectomy (LT) and TT was performed in 11.5% and 88.5%, respectively. Recurrent laryngeal nerve paralysis and hypoparathyroidism was less commonly observed in LT (1.3% and 0%) than TT (2.4% and 3.5 %). Fifty‐six cases (1.4%) had distant recurrence. Recurrence‐free survival rates at 10 years was 93.5%. There was no significant difference in recurrence rate between LT and TT. Age ≥55, cN1b, and tumor diameter >30 mm significantly associated with distant recurrence. There was a strong relationship between the number of positive risk factors and recurrence; the distant recurrence rate in cases of 0, 1, 2, and 3 positive factors was 0.3% (4/1203), 1.3% (25/1889), 2.7% (23/830) and 7.1% (4/52) (HR 6.46 (2.34–17.86), Log‐rank <0.001).

**Conclusion:**

For intermediate‐risk conventional PTC, there is no difference in prognosis even if LT was selectively conducted. However, in patients with risk factors for distant metastatic recurrence, such as age ≥55 years, cN1b, and tumor size >30 mm, adjuvant RAIT was considered eligible.

## 1 INTRODUCTION

Patient with papillary thyroid carcinoma (PTC) is classified into low‐, high‐ and intermediate‐risk based on the disease status at the time of initial presentation, depending on the risk of recurrence or cancer death, and risk‐oriented management is recommended as a standard treatment of choice.^1^, ^2^ Low‐risk patients are generally treated with surgery alone, and postoperative adjuvant treatment with radioactive iodine treatment (RAIT) is not recommended. On the other hand, RAIT is normally added for adjuvant or treatment purpose after total thyroidectomy (TT) for high‐risk patients. For intermediate‐risk patients, TT followed by RAIT was basically recommended as a standard treatment strategy. However, several recent studies found less aggressive treatment, for example, lobectomy (LT) or TT without RAIT, showed no inferiority in intermediate‐risk PTC patients' outcome.^3^, ^4^, ^5^, ^6^ The main purpose of adjuvant RAIT is to prevent life‐threatening recurrence, such as distant metastasis, and improve survival. However, it is uncertain whether adjuvant RAIT can additionally improve prognosis of intermediate‐risk PTC patients.^7^ The intermediate‐risk PTC patient includes those with cancers of wide varieties of grades of progression with different malignant potentials. Therefore, it is difficult to recommend treatment strategies uniformly to this group of patients. Furthermore, many previous reports were based on retrospective analysis, and the study results were largely influenced by inclusion criteria of the patient selected for consideration and by case‐by‐case treatment that had been performed.

The purpose of this study is to identify factors that pose a risk of distant metastatic recurrence after initial surgery from a retrospective analysis using a large number of patients of intermediate‐risk conventional PTC consequently treated by standardized protocol without RAIT, in an aim to determine predictive factors that could indicate appropriate candidates for adjuvant RAIT.

## 2 PATIENTS AND METHODS

### 2.1 Patients

Fifteen thousand and forty‐one PTC patients who underwent initial surgery at Kuma hospital between 2005 and 2022 were enrolled. The patients were classified according to the UICC/AJCC TNM classification (eighth edition).^8^ Patients with postoperative pathologically proven conventional type PTC were chosen. Patients with uncertain information of preoperative cytological diagnosis, tumor diameter, TNM status, and prognosis were excluded. We additionally excluded low‐ (T1N0M0) and high‐risk cases (either of tumor over 4 cm in diameter, gross extrathyroidal extension, N1 with extra nodal extension, N1 with any lymph node >3 cm or M1) based on Japanese guideline^2^ by preoperative and intraoperative findings, and patients with coexisting cancers of histological types other than conventional type PTC. Finally, 4030 intermediate‐risk PTC patients were selected and retrospectively examined the outcomes. Overall survival (OS) was defined as the period between initial operation and the time of death by any reason. Cause specific survival (CSS) was defined as the period between initial operation and the time of death by thyroid cancer. Recurrence free survival (RFS) was defined as the period between initial operation and the time of identifying recurrence in any part of the body (IRB approval 20200709‐1).

### 2.2 Surgical treatment strategy

At our institute, TT has been chosen as the standard surgical procedure for intermediate‐risk PTC patients. If lymph node metastasis was not evident preoperatively (cN0), dissection of the central compartment on both sides (D1) was performed, and if metastasis was present (cN1), lateral node dissection on the affected side (D2) was added. In case there was tumor invasion into the strap muscles (T3b), we add D2 on the affected side. However, LT could be selected based on the patient's preferences, and D1 was selected in cases when lymph node metastasis was not suspected by ultrasound examination or where ultrasound‐guided lymph node fine needle aspiration cytology (FNA) specimen did not demonstrate cancer cells or elevated thyroglobulin (Tg) was not proved in the washing fluid of the needle.

### 2.3 Follow‐up

Physical examination and blood tests were performed 1, 3, and 6 months after surgery. Thyroid functional supplementation therapy with levothyroxine was initiated soon after TT or if deemed necessary in LT cases by the attending physician. In cases with supplementation therapy, TSH level below normal range was determined ideal during follow‐up period. Vocal cord movement was investigated using a laryngeal fiberscope within 1 week after surgery, and if any abnormalities were found, re‐examination was performed 1, 3, and 6 months later. Persistent paralysis was defined when vocal cord was still fixed at the 6‐month examination. Serum PTH was measured on the day after surgery, and was repeated at 1, 3, and 6 months later when needed. If the serum PTH level showed below normal and calcium or vitamin D supplementation was still required at 6 months from operation, the patient was diagnosed with persistent hypoparathyroidism. Cervical ultrasound examinations were performed annually. CT scans of the chest were conducted when it was necessary, for example, when elevated level of thyroglobulin (Tg) was found. Recurrence was diagnosed when structural disease was confirmed either by imaging studies or by FNA.

### 2.4 Statistics

EZR was used for statistical analysis. A two‐tailed Fisher's exact test was used to compare the incidences of two groups, and distant RFS times were compared using the Kaplan‐Meier method using the Log‐rank test. Cox proportional hazards model was used to analyze factors affecting prognosis. A *p*‐value < 0.05 was determined as statistically significant.

## 3 RESULTS

The characteristics of the patient was demonstrated in Table 1. Females, older patients, tumors <30 mm were more often found. LT and TT was performed in 465 (11.5%) and 3565 (88.5%) patients, respectively. LT was chosen significantly more frequently in patients aged 55 years or older and in patients with cT3b, cN0, and cN1a disease (data not shown). D2 was omitted in some cases without lateral nodal involvement and D1 and D2 dissection was performed in 2132 (52.9%) and 1898 (47.1%), respectively. Thyroid hormone supplementation was needed in 53.0% of LT and all TT cases. Persistent recurrent laryngeal nerve paralysis and hypoparathyroidism was observed in 1.3% and 2.4% (*p* = 0.180), and in 0% and 3.5% (*p* < 0.001) of LT and TT cases, respectively.

**TABLE 1:** Characteristics of the patients and the frequency of distant recurrence.

Category	*n*	%	Distant recurrence	%	*p*
Sex					
Female	3269	81.10%	46	1.41%	1.000
Male	761	18.90%	10	1.31%	
Age	7–90 (median 49.0) years				
<55	2484	61.60%	23	0.93%	0.002
≧55	1546	38.40%	33	2.13%	
Tumor diameter	2–40 (median 22.0) mm				
30 mm or smaller	3388	84.07%	41	1.21%	0.040
>30 mm	642	15.93%	15	2.34%	
cT category					
1	1674	41.50%	23	1.37%	1.000
2	2217	55.00%	31	1.40%	
3b	139	3.40%	2	1.44%	
cN category					
0	1483	36.80%	10	0.67%	0.003^a^
1a	947	23.50%	11	1.16%	
1b	1600	39.70%	35	2.19%	0.001^b^
cStage					
I	2866	71.10%	31	1.08%	0.011
II	1164	28.90%	25	2.15%	
Thyroidectomy					
Total (TT)	3565	88.50%	54	1.51%	0.058
Less than total (LT)	465	11.50%	2	0.43%	
Nodal dissection					
Central only (D1)	2132	52.90%	15	0.70%	<0.001
Central and lateral (D2)	1898	47.10%	41	2.16%	
Total	4030	100%	56	1.39%	

^a^ cN0 versus cN1a and cN1b.

^b^ cN1b versus cN0 and cN1a.

**FIGURE 1:**
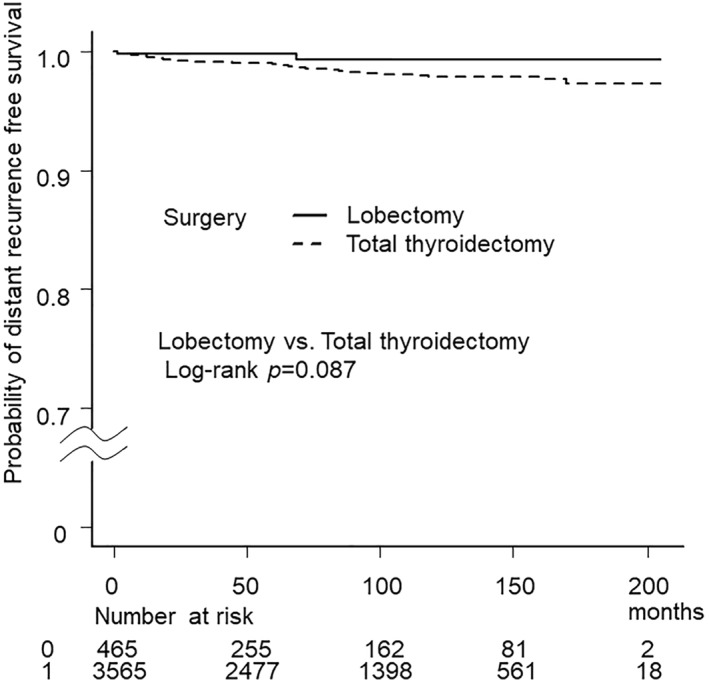
Distant recurrence free survival according to the surgical method. There was no difference in distant recurrence free survival rate between patients who underwent lobectomy and total thyroidectomy.

During the follow‐up period (6 to 205; median 83.7 months), 191 cases (4.7%) showed recurrence. RFS rates at 5, 10, 15 years was 96.5%, 93.5%, 90.8%, respectively. Distant recurrence was found in 56 patients (1.4%). Fifty patients had lung metastasis and six had bone metastasis. The distant RFS was not worse in patients who chose LT compared to those chose TT (Figure 1).

Distant recurrence was more often found in patients with older age (55 and over), with larger tumor (larger than 30 mm), showed cN1, had lateral nodal involvement (cN1b), showed advanced clinical stage, underwent D2 dissection (Table 1). There was a significant confounding between cN1b and cN1, D2 as well as clinical stage. Multivariate analysis revealed patients with older age, who had larger tumor, showed cN1b were the independent factors to indicate distant recurrence (Table 2).

**TABLE 2:** Multivariate analysis concerning distant metastasis.

Factors	Odds ratio (95% confidence interval)	*p*
Lateral node involvement (cN1b)	2.71 (1.49–4.93)	0.001
Age 55 or over	2.50 (1.46–4.30)	<0.001
Total thyroidectomy	2.27 (0.54–9.64)	0.266
Tumor diameter >30 mm	2.18 (1.08–4.38)	0.029
T2 or T3b	1.17 (0.60–2.28)	0.637
Male	0.79 (0.39–1.58)	0.497

**FIGURE 2:**
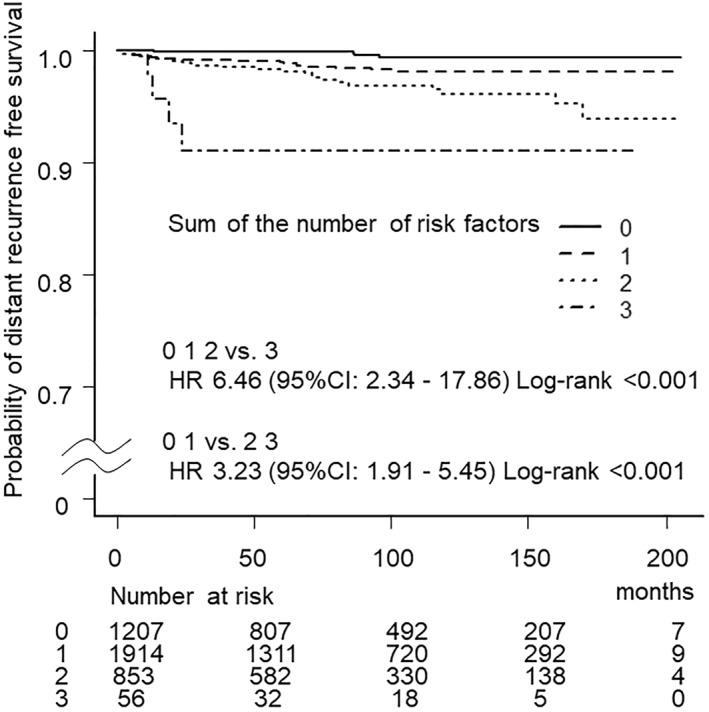
Correlation between the number of positive risk factors and the rate of distant recurrence. There is a strong correlation between the number of positive risk factors and the rate of distant recurrence. A significantly higher distant recurrence rate was found in cases who had all three positive risk factors (HR 6.46 (2.34–17.86), Log‐rank <0.001).

There was a strong correlation between the number of positive risk factors and the rate of distant recurrence; the distant recurrence rate in cases who had 0, 1, 2, and 3 positive risk factors was 0.3% (4/1203), 1.3% (25/1889), 2.7% (23/830) and 7.1% (4/52) (HR 6.46 (2.34–17.86), Log‐rank <0.001) (Table 3 and Figure 2).

**TABLE 3:** Correlation between number of positive risk factors and distant metastasis.

Number of positive risk factors^a^	Total number of patients	Number of patients with distant recurrence	%
0	1207	4	0.3%
1	1914	25	1.3%
2	853	23	2.7%
3	56	4	7.1%

^a^ Risk factors: Age 55 or over, Tumor diameter >30 mm, Clinical lateral nodal involvement.

There were 81 deaths (2.0%). Ten patients (0.2%) died of cancer, 60 (1.5%) died of other causes, and the cause of death was unknown in 11 patients. Of the patients died of cancer, 9 patients had local lesions and 4 patients had distant metastases (3 had both). The OS rates at 5, 10, and 15 years after surgery were 99.1%, 97.2%, and 93.8%, and the CSS rates were 99.8%, 99.6%, and 99.6%, respectively. In both univariate and multivariate analysis, OS was significantly worse in patients who were older, male, and had large tumor diameter. Disease‐specific mortality was observed only in patients who were older and underwent TT. There were no significant differences in OS or DSS due to different surgical methods.

## 4 DISCUSSION

The risk classification system for PTC patients in Japan^2^ is basically similar as that described in the ATA guidelines.^1^ However, some patients who are classified as intermediate‐risk in the ATA guidelines^1^ due to postoperative histological findings such as vascular invasion, five or more lymph node metastases, high‐grade histological findings, or microscopic extrathyroidal extension are not classified as intermediate‐risk in the Japanese guideline.^2^ On the other hand, some patients with intrathyroidal PTC measuring 2–4 cm in diameter and patients with fewer than five lymph node metastases are classified as intermediate‐risk. This is because adjuvant RAIT is usually omitted for intermediate‐risk patients in Japan. Therefore, TT is not performed in all intermediate‐risk patients, and the extent of thyroidectomy is decided at the discretion of the attending physician, considering preoperative prognostic factors and patient background.^2^ Previous reports have confirmed that intermediate‐risk PTC patients demonstrate a good DFS^9^ or CSS^10^ using these refrained treatment concepts regardless surgical method or without adjuvant RAIT in Japan. These results were not inferior to those reported in other part of the world. The main cause of death in intermediate‐risk PTC patient was death from other diseases, as found in the present study. Death of cancer was rare at 0.2%, and disease recurrence (found in 4.7% of the patients) was less likely to cause death directly. The only determining factor for DSS was older age, as have been demonstrated.^10^, ^11^

In principle, we performed TT for intermediate‐risk PTC, but LT was allowed if patient desired. As a result, LT was performed in 12% of cases, mainly in cases where cN1b was not proven preoperatively. No worsening of RFS, OS or CSS was observed compared to TT cases in the present study. Similar results have been reported using a large database.^3^ Approximately half of LT cases did not require postoperative thyroid hormone supplementation, and recurrent laryngeal nerve paralysis and hypoparathyroidism were less common than in TT, as demonstrated in the present study. LT was, thus, considered acceptable in cases that could be judged to have no poor prognostic factors. Contrarily, in intermediate‐risk PTC cases with the risk factors for distant metastasis, TT should be performed with the addition of postoperative adjuvant RAIT in mind.

In previous studies, the recommendation on whether to perform RAIT as adjuvant therapy to the patients with intermediate‐risk PTC differs from study to study. Because each report investigated patients with different characteristics, even though they were stratified to intermediate‐risk PTC. Additionally, Wang^12^ discussed the hypotheses to explain these inconsistent results, including (1) ethnic difference, (2) too short follow‐up period, (3) difference in surgical protocol.

A retrospective analysis, using 21,870 patients form National Cancer Database, reported that adjuvant RAI therapy improved survival in patients with intermediate‐risk PTC. This study uses OS for the outcome.^13^ As seen in this study, the main cause of death in intermediate‐risk PTC was not the disease itself and less than half patients were died of disease. Therefore, it is necessary to consider the difference of analyzing OS and DSS when evaluating the efficacy of RAIT. Tian et al. found that RAIT in 1487 intermediate‐risk PTC patients with low postoperative Tg levels had a significant benefit in decreasing the rate of local recurrence with HR of 10.572.^14^ On the other hand, Kim et al. found that RAIT was not related preventing loco‐regional recurrence by investigating 8297 intermediate‐risk PTC patients undergone TT.^5^ A meta‐analysis including 56,266 intermediate‐risk PTC patients could not demonstrate the benefit of RAIT to improve RFS, either.^6^

At the same time, several reports demonstrated that there is certain a group of intermediate‐risk PTC patients for whom RAIT demonstrated a positive benefit on survival. A recent study showed RAIT improved DSS in intermediate‐risk PC patient by using 23,107 cases from SEER database. The study demonstrated the benefit was limited in patients with male sex, age 45 or older, and tumor size 20 mm or larger.^12^ Nixon demonstrated the criteria for omitting RAIT in intermediate‐risk PTC patient, such as those who had smaller tumor less than 40 mm that confined to the thyroid gland and without lymph node metastasis.^15^ Our observation in the present study demonstrated risk factors for distant metastasis; age 55 or over, tumor diameter larger than 30 mm and clinical lateral nodal involvement were in line with these observations. In addition, we showed that the accumulation of these factors increased the risk for distant recurrence. These factors could easily be determined before conducting initial surgical treatment. We believe our results give a convenient way to choose patient that should necessarily treated with TT followed by adjuvant RAIT among conventional type intermediate‐risk PTC patients.

Limitations of this study include (1) the presence of bias in selecting the surgical method for each case due to retrospective study, (2) many cases was dropped due to changes in general rules for the description of thyroid cancer in Japan or due to missing records, (3) treatment methods after recurrence have not been investigated, such as RAIT or TSH suppression status, (4) the risk classification of PTC used in this study is not completely consistent with that of ATA guidelines. However, this study, which showed the long‐term prognosis of a large number of intermediate‐risk conventional PTC cases, could contribute to the formulation of future treatment policies.

## 5 CONCLUSION

For intermediate‐risk conventional PTC, there was no difference in prognosis even if LT was conducted. LT showed fewer complications than TT and would be beneficial for patients if no difference in prognosis was observed. However, in patients with risk factors for distant metastatic recurrence, such as age ≥55 years, cN1b, and tumor size >30 mm, adjuvant RAIT may be considered.

## AUTHOR CONTRIBUTIONS

**Naoyoshi Onoda**: Conceptualization; data curation; formal analysis; investigation; methodology; project administration; writing – original draft; writing – review & editing. **Yasuhiro Ito**: Writing – review & editing. **Akihiro Miya**: Writing – review & editing. **Minoru Kihara**: Writing – review & editing. **Akira Miyauchi**: Supervision; writing – review & editing.

## CONFLICT OF INTEREST STATEMENT

The authors declare no conflicts of interest.

## ETHICS STATEMENT

This study was approved by institutional ethics committee (# 20200709‐1).
